# Effectiveness of the Home Based Life Saving Skills training by community health workers on knowledge of danger signs, birth preparedness, complication readiness and facility delivery, among women in Rural Tanzania

**DOI:** 10.1186/s12884-016-0916-x

**Published:** 2016-06-02

**Authors:** Furaha August, Andrea B. Pembe, Rose Mpembeni, Pia Axemo, Elisabeth Darj

**Affiliations:** Department of Obstetrics and Gynaecology, Muhimbili University of Health and Allied Sciences, Dar es Salaam, Tanzania; Department of Women’s and Children’s Health, International Maternal and Child Health, Uppsala University, Uppsala, Sweden; Department of Epidemiology and Biostatistics, School of Public Health and Social Sciences, Muhimbili University of Health and Allied Sciences, Dar es Salaam, Tanzania; Department of Public Health and General Practice, Norwegian University of Science and Technology, Trondheim, Norway

## Abstract

**Background:**

In spite of government efforts, maternal mortality in Tanzania is currently at more than 400 per 100,000 live births. Community-based interventions that encourage safe motherhood and improved health-seeking behaviour through acquiring knowledge on the danger signs and improving birth preparedness, and, ultimately, reduce maternal mortality, have been initiated in different parts of low-income countries. Our aim was to evaluate if the Home Based Life Saving Skills education by community health workers would improve knowledge of danger signs, birth preparedness and complication readiness and facility-based deliveries in a rural community in Tanzania.

**Methods:**

A quasi-experimental study design was used to evaluate the effectiveness of Home Based Life Saving Skills education to pregnant women and their families through a community intervention. An intervention district received training with routine care. A comparison district continued to receive routine antenatal care. A structured household questionnaire was used in order to gather information from women who had delivered a child within the last two years before the intervention. This questionnaire was used in both the intervention and comparison districts before and after the intervention. The net intervention effect was estimated using the difference between the differences in the intervention and control districts at baseline and endline.

**Results:**

A total of 1,584 and 1,486 women were interviewed at pre-intervention and post intervention, respectively. We observed significant improvement of knowledge of three or more danger signs during pregnancy (15.2 % vs. 48.1 %) with a net intervention effect of 29.0 % (95 % CI: 12.8–36.2; *p* < .0001) compared to the comparison district. There was significant effect on the knowledge of three or more danger signs during childbirth (15.3 % vs. 43.1 %) with a net intervention effect of 18.3 % (95 % CI: 11.4–25.2; *p* < .0001) and postpartum for those mentioning three or more of the signs (8.8 % vs. 19.8 %) with net effect of 9.4 % (95 % CI: 6.4–15.7; *p* < .0001). Birth preparedness practice improved for those who made more than three actions (20.8 vs. 35.3 %) with a net intervention effect of 10.3 % (95 % CI: 10.3–20.3; *p* < .0001) between the intervention and control district at pre-intervention and post intervention. Utilisation of antenatal care with four visits improved significantly (43.4 vs. 67.8 %) with net effect of 25.3 % (95 % CI: 16.9–33.2; *p* < .0001), use of facility delivery improved in the intervention area (75.6 vs. 90.2 %; *p* = 0.0002) but there was no significant net effect 11.5 % (95 % CI: -5.1–39.6; *p* = 0.123) compared to comparison district.

**Conclusion:**

This study shows that a community-based intervention employing community health workers as teachers in delivering Home Based Life Saving Skills program to pregnant women and their families improved their knowledge of danger signs during pregnancy, childbirth and postpartum, preparedness for childbirth and increased deliveries at health facilities which employ skilled health workers in this rural community.

**Electronic supplementary material:**

The online version of this article (doi:10.1186/s12884-016-0916-x) contains supplementary material, which is available to authorized users.

## Background

Millennium development goal number (MDG) five proposed that, by 2015, maternal mortality should be reduced by 75 % from that of the level reported in 1990. To date there has been progress showing that maternal deaths are declining worldwide by 45 %, however, this is occurring much more slowly in Sub-Saharan countries [1]. Barriers to achieve MDG 4 and 5 among other things include lack of government funding and political will, barriers to accessing health care such as distance, few skilled attendants and lack of quality care, poor-functioning health systems [2]. Improving health systems, reducing inequities in maternal and sexual reproductive health and availability of quality of care will contribute towards achieving the sustainable development goals [3]. In spite of government efforts, maternal mortality in Tanzania is currently more than 400 per 100,000 live births [4].

Evidence-based interventions have addressed this challenge, including providing skilled attendance for antenatal and delivery care as well as counselling on danger signs, birth preparedness and complication readiness (BP/CR) plans and provision of emergency obstetric care [5]. Accessibility to skilled care and other maternal health services is hampered by poverty, lack of knowledge and education, traditional norms, lack of skilled care and poor quality of care [6–9]. Community-based interventions have been initiated in different areas of low-income countries to improve accessibility to maternal health services, but still many women do not deliver in health facilities [10–12].

It is expected that knowledge of danger signs in the community can help in reducing the delays in seeking care in case of an emergency and in accessing care and hence reduces the risk of maternal morbidity and mortality. Evidence from studies done in Tanzania, Ethiopia and Uganda show that knowledge of danger signs during pregnancy, delivery and postpartum is low in rural communities [13–16]. Moreover it has also been demonstrated that few women in Tanzania are counseled on the danger signs during their antenatal visits [17, 18], despite the government having proposed individualised antenatal care counselling through Focused Antenatal Care (FANC) guidelines [19]. There is a need to improve the awareness of danger signs in the community and eventually increase demand for care through other strategies such as community-based interventions.

In addition, BP/CR plans, together with knowledge of danger signs, are advocated in order to improve community awareness and readiness for normal delivery and obstetric emergencies [20, 21]. Previous studies have shown that health workers assisting women with birth preparedness plans in the health facility led to increased utilisation of skilled care for delivery [22, 23]. However, few studies have been conducted at community level to evaluate the impact of community-based interventions, particularly those that use the concept of Home Based Life Saving Skills (HBLSS) on knowledge of danger signs, birth preparedness, complication readiness and facility delivery.

HBLSS is a community-based training program developed by the American College of Nurse Midwives (ACNM) [24]. This training is provided to pregnant women together with immediate family members with the aim of recognising life-threatening conditions, promoting health-seeking behaviour, birth preparedness and complication readiness, using life-saving skills to stabilise the patient when a problem occurs at home. HBLSS is conducted through story-telling, role-playing and skill acquisition using pictorial cards called Take Action Cards (TAC).

HBLSS was introduced in Tanzania in 2007 where 24 health workers from various parts of the country were trained by ACNM consultants. The 24 health personnel became master trainers. The training was provided in collaboration with the White Ribbon Alliance as advocacy in addressing the challenges of maternal mortality in Tanzania. The Ministry of Health and Social Welfare supported the training. The master trainers then went on to train HBLSS trainers and they eventually trained HBLSS guides. The HBLSS guides were mainly health attendants who were working at health facilities. The HBLSS guides trained pregnant mothers together with other family members on HBLSS modules.

While the HBLSS program is designed to be delivered in the community by health professionals, such as midwives, nurse attendants or health attendants, in this study we trained community health workers as HBLSS guides instead. HBLSS training at community level has not yet been evaluated as a community-based strategy in a rural area in Tanzania.

Our aim was to evaluate whether HBLSS training by community health workers would improve knowledge of danger signs, birth preparedness, complication readiness and facility-based deliveries in a rural community in Tanzania.

## Methods

### Study setting

The study was conducted in the Rufiji and Mkuranga districts in the Pwani Region located in the eastern part of Tanzania. Rufiji district has a population of 217,274, and Mkuranga district has 222,921 [4]. The majority of the population in these districts live below the poverty line and most are subsistence farmers [4]. The literacy rate in the Pwani region among females is 66.9 %. The Rufiji district has two hospitals, four health centers and 54 dispensaries, while the Mkuranga district has one hospital, five health centers and 53 dispensaries. The hospitals provide emergency obstetric care and other reproductive health services while the health centers and dispensaries provide antenatal care (ANC) services, cater for normal deliveries and refer complicated obstetric cases to the hospitals. The Rufiji district was chosen as the intervention district because of previous studies completed in the area demonstrating that pregnant women had a low awareness of danger signs [14]. Mkuranga was chosen as a comparison district due to its comparable population size and socio-demographic characteristics.

### Study participants

Inclusion criteria for participation, both in the intervention and control districts, were to be women who had delivered a child in the last two years before the survey questionnaire was administered pre-intervention. The same criteria were applied for women who answered the questionnaire post intervention. As this was not a longitudinal study, the participants who took part in the pre intervention survey were not necessarily the same as those in the post intervention survey.

### Study design

**Fig. 1:**
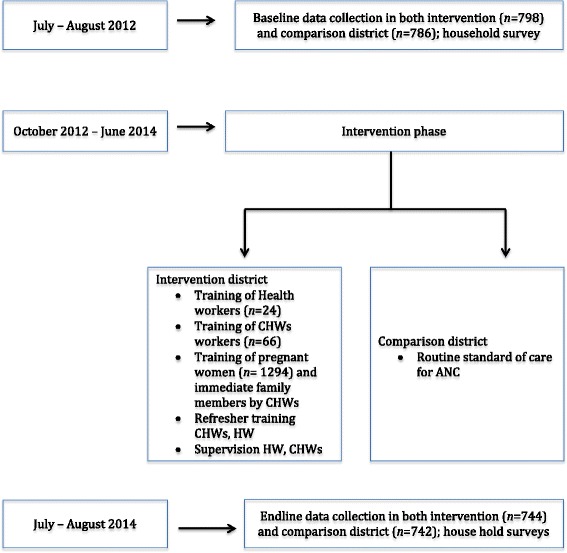
Phases of the study and data collection

This was a quasi-experimental study (non-equivalent group) using pretest-posttest comparison to evaluate the effects of HBLSS training in the community on knowledge of obstetric danger signs, BP/CR and facility delivery (Fig. 1).

### Sample size and sampling

A two-stage cluster sampling strategy was employed to select a representative sample from the two districts. First, all health facilities were listed and then 14 health facilities in each district were randomly selected using the ballot method. In Rufiji district four health centres and ten dispensaries were selected while in Mkuranga district five health centres and nine dispensaries were selected. Secondly, two villages, belonging to these health facilities were randomly selected. From these villages all women who had delivered in the last two years were selected for interview. The estimated sample size was 1,400 (700 per district) based on the facility delivery of 47 % in Tanzania [25] with the assumption of detecting a 15 % effect on increased facility delivery as the primary outcome, the power of 90 % with a 5 % significant level, and assuming a 3 % non-response rate. The same villages in both districts were visited before and after the intervention.

### Intervention

The HBLSS programme includes 12 modules that cover core topics for women and family members, maternal complication and newborn complications. The TAC cards represent pictures that show different problems on one side and what action to take on the other. Family members are given the card to keep in their home at the end of the sessions. The intervention period lasted 21 months.

#### Training of community health workers

A master trainer (the first author) was trained by ACNM consultants and subsequently trained 24 health workers for one week from selected health facilities in the intervention district. The health workers included nurse midwives and nurse attendants. The health workers were given education on the HBLSS curriculum and they, in turn, trained 66 community health workers (CHW) for two weeks. The CHWs were selected from their respective villages in the catchment population. The selection criteria included being a member of the village and being able to read and write. The CHWs usually work on voluntary basis and help to provide public health interventions, such as vaccination programs, health education activities, and outreach services. In this project the CHWs provided education to the community using HBLSS without affecting provision of other preventive and health promotion services. The CHWs were provided with monthly allowance of Tanzanian shillings 30,000 (USD $13.60).

#### Teaching the community

After completing the training the CHWs were required to identify pregnant women in the community in the catchment village. Upon this identification, the CHWs were supposed to make at least four visits to the women during their pregnancy. During the home visits they provided education on the HBLSS modules and health promotion messages to the currently pregnant woman in the presence of immediate family members and husbands. The topics included recognition of danger signs during pregnancy, delivery and postpartum, as well as neonatal danger signs. Additionally, topics about preparation for birth, antenatal care attendance and the promotion of health-seeking behaviour to avoid maternal deaths were discussed. The teaching included the use of stories, discussions and skills acquisition using checklists. Information about the home visits was routinely recorded by the CHWs. The CHWs made arrangements in advance for the convenient time when the pregnant woman, husband and other family members would be available for the sessions.

#### Supervision

Health workers checked the records collected by CHWs upon completion, and acted as the immediate supervisors of the CHWs. Each health worker supervised two to four CHWs. Health workers also made occasional visits to the CHWs during home visits to monitor their progress. These visits were unannounced and the health workers occasionally participated in the provision of the education during some of the visits to the women’s homes. The visits took place once a month for each CHW. During these visits they would discuss challenges related to the education program as well as strategies to tackle the challenges. The Master trainer supervised the health workers and participated in some of the education meetings. A one-day refresher training session was provided to the health workers and CHWs every two months.

The comparison district continued to receive the usual services provided at the health facilities by health workers.

### Data collection

A household pre-intervention survey was conducted in the intervention area and the comparison area from July to August 2012. An endline survey was conducted from July to August 2014. Seven experienced interviewers, who were medical doctors, were trained and collected data under the supervision of the first author. The questionnaire was piloted with 12 women and was slightly adjusted before data collection began. The questionnaire was used to collect information on socio-demographic characteristics (age, marital status, education level), obstetric characteristics (parity, number of ANC visits, place of delivery), knowledge of danger signs (during pregnancy, childbirth and postpartum), and knowledge and practice of birth preparedness components. The questionnaire was adapted from the John Hopkins Program for International Education in Gynecology and Obstetrics (JHPIEGO) questionnaire on BP/CR [26] (Additional file 1). The outcome variable for place of delivery was coded as 1 for health facility delivery and 0 for home delivery.

Key obstetric danger signs were divided into three groups; during pregnancy, delivery, and the postpartum period.

Participants were asked to mention any danger signs they were aware of during pregnancy, delivery and in the postpartum period, without being given any options. The research assistant would tick off the danger signs that the participant mentioned on the questionnaire. Possible options were: during *pregnancy*: excessive vaginal bleeding, swollen face/hands, blurred vision, severe headaches, fits, and severe abdominal pain.

In the *delivery phase*, danger signs included: excessive vaginal bleeding, severe headache, labour lasting more than 12 h, severe abdominal pain, fits and retained placenta.

In the *postpartum phase* the danger signs were: excessive vaginal bleeding after delivery, severe headache, fits, fever and foul-smelling discharge. Knowledge of any danger sign during any of the three phases was coded yes or no.

*Knowledge* of birth preparedness was coded yes or no if one could mention any of the six birth preparedness components: saving money, identifying transport, identifying skilled attendant, identify where to go in case of emergency, identifying blood donor, and identifying birth kits. Identifying birth kit is not part of the BP/CR by JHPIEGO but was included as it was mentioned as a common preparation method during the pretest of the questionnaire. Knowledge of three or more of the components scored the woman as being knowledgeable.

*Practice* for birth preparedness was coded yes or no if a woman made any of the following preparations in her last pregnancy: saved money, identified transport, identified skilled attendant, identified where to go in case of emergency, identified blood donor, and identified birth kits. Likewise, a score of three or more practiced components were defined as acceptable birth preparedness in our study.

The household wealth quintiles were calculated using asset ownership. This was estimated using principal component analysis (PCA) [27]. Items included in the asset index included were: owning a radio, owning a bicycle, owning a mobile phone, type of floor material, source of drinking water and source of cooking fuel. The sample was divided into five quintiles with A1 being poorest (20 % of the participants) and A5 being least poor (20 % of the participants). Questionnaires were thoroughly checked by the team on a daily basis to determine whether they had been completed correctly. If information was missing, the women were contacted to obtain the lacking data.

### Outcome measures

The primary outcome measure was proportion of women who delivered in a health facility. Secondary outcome measures were utilization of skilled care for ANC for four visits, knowledge of at three danger signs during pregnancy, childbirth and postpartum, knowledge of three BP/CR components and actions taken for BP/CR.

### Data analysis

Data were entered into SPSS and cleaned, and in case of any discrepancies, the original completed questionnaire was used for cross-checking. Descriptive statistics were used to describe survey respondents and their characteristics. For each group (pre/post intervention) and time point (baseline/endline) an estimated proportion of each outcome variable and its variance was calculated according to the cluster sampling design [28].

The net intervention effect (NIE) was estimated as the difference between intervention and comparison groups regarding changes in proportions from baseline to endline (difference between intervention and comparison groups before (baseline) and after (endline) the intervention). In addition, a comparison of the post-intervention difference was completed. This effect is a linear combination of four independent estimates. *P*-values from a *Z*-test and 95 % confidence intervals for the intervention effect were calculated based on a normal distribution assumption. *P* < 0.05 was considered a statistically significant result. Statistical analyses were performed with SAS version 9.4 (SAS Institute Inc., Cary, NC, USA).

## Results

In the intervention district the CHWs trained 1,294 pregnant women, 1096 men and 766 family members. In the post-intervention survey, 96.2 % of women reported to have been visited once, 73.5 % twice, 62.5 % three times, and 47.3 % four times by the CHWs. Characteristics of the participants in the intervention and comparison district were comparable with no significant difference in terms of age, marital status, education level and parity. There was no difference across the asset quintiles in the intervention and comparison districts (Table 1).

**Table 1:** Background characteristics of women in Rufiji and Mkuranga districts at Pre-intervention and Post-intervention

	Pre intervention			Post intervention		
	*n* = 798	*n* = 786		*n* =744	*n* =742	
	Intervention *n* (%)	Comparison *n* (%)	*p value*	Intervention *n* (%)	Comparison *n* (%)	*p value*
Age of participants			0.463			0.725
< 21	177(22.2)	169(21.5)		139(18.7)	178(24.0)	
21–25	228(28.6)	213(27.1)		193(25.9)	166(22.4)	
26–30	181(22.7)	192(24.4)		171(23.0)	166(22.4)	
31–35	128(16.0)	120(15.3)		119(16.0)	109(14.7)	
> 35	84(10.5)	92(11.7)		122(16.4)	123(16.6)	
Marital status			0.652			0.858
Single	173(21.7)	119(15.1)		152(20.4)	139(18.7)	
Married/Cohabiting	625(78.3)	667(84.9)		592(79.6)	603(81.2)	
Education level			0.351			0.731
No school	217(27.2)	262(33.3)		227(30.5)	258(34.8)	
Primary incomplete	94(11.8)	68(8.7)		62(8.1)	54(7.3)	
Primary completed	445(55.9)	420(53.4)		390(52.4)	376(50.7)	
Secondary and higher	40(5.1)	36(4.6)		6 5(8.7)	54(7.3)	
Missing	2					
Asset quintile			0.613			0.348
A1 poorest	22.7	18.1		23.7	19.6	
A2	21,2	23.2		19.1	22.8	
A3	21,2	18.5		19.8	18.3	
A4	18.1	21.0		17.0	20.8	
A5 least poor	16.8	19.2		20.4	18.5	
Obstetric complication	144(18.0)	130(16.5)	0.872	132(17.7)	103(13.9)	0.761
Parity			0.647			0.849
1	218(27.3)	162(20.6)		199(26.7)	184(24.8)	
2–4	409(51.3)	432(55.0)		354(47.6)	364(49.1)	
> 4	171(21.4)	196(24.4)		191(25.7)	194(26.1)	

Table 2 shows the effect of the intervention on maternal services utilisation. There was a statistically significant increase in institutional deliveries in the intervention district from 75.6 to 90.2 % as compared to the comparison district, which increased from 76.1 to 79.6 %. The increase was higher in the intervention group, but the net effect was not statistically significant comparing the two groups 11.5 % (95 % CI: -5.1–39.6; *p* = 0.123). There was a significant increase in attending more than four ANC visits in the intervention area (43.6 % vs. 67.8 %) compared to the comparison area with a net effect of 25.3 % (95 % CI: 16.9–33.2; *p* < .0001).

**Table 2:** Effect of the intervention on facility delivery among women in Rufiji and Mkuranga districts

	Pre intervention			Post intervention			Difference		
	*n* = 798	*n* = 786		*n* = 744	*n* = 742				
	Intervention *n* (%)	Comparison *n* (%)	Difference (%)	Intervention *n* (%)	Comparison *n* (%)	Difference (%)	NIE (%)	95 % CI	*p-*value
Facility delivery	603 (75.6)	598 (76.1)	-0.5	671 (90.2)	588 (79.2)	11	11.5	-5.1-39.6	0.123
ANC visits									
At least 1	735 (92.1)	765 (93.5)	-1.5	717 (96.4)	697 (94.0)	2.2	3.7	-1.1-5.6	0.119
4 or more	335 (42.4)	339 (43.2)	-0.8	504 (67.8)	322 (43.4)	24.4	25.2	16.9 -33.2	< .0001

Table 3 shows the effect of the intervention on maternal *knowledge* of danger signs, which had improved. Significantly more women could mention three or more danger signs during pregnancy after the intervention with a net effect of 29.0 % (95 % CI: 12.8–36.2; *p* < .0001) compared to the comparison district. Likewise there was effect on the knowledge of danger signs during childbirth with a net effect of 18.3 % (95 % CI: 11.4–25.2; *p* < .0001) and postpartum for those mentioning three or more of the signs 9.4 % (95 % CI: 6.4–15.7; *p* < .0001).

**Table 3:** Effect of the intervention on knowledge of danger signs among women in Rufiji and Mkuranga districts

	Pre intervention (*N* = 798)			Post intervention			Difference		
	(*n* = 798)	(*n* = 786)		(*n* = 744)	(*n* = 742)				
Knowledge	Intervention (%)	Comparison (%)	Difference (%)	Intervention (%)	Comparison (%)	Difference (%)	NIE (%)	CI 95 %	*p-*value
During Pregnancy									
Heavy Bleeding	14.7	15	0.3	41.3	15.5	25.8	25.5	11.6–32.8	< .0001
Fever	19.7	17.9	1.8	30.2	23.6	6.6	4.8	-1.5–11.8	0.078
Fits	14.4	17.3	-2.9	26.5	15.8	10.7	13.6	10.7–20.2	< .0001
Headache	5.1	5.7	0.6	10.3	3.4	6.7	6.1	3.8–11.5	< .0001
Knowledge of at least 3 (Out of 12)	15.2	18.5	-3.3	48.1	22.4	25.7	29.0	12.8–36.2	< .0001
During Childbirth									
Bleeding	24.9	24.4	0.5	53.1	34.5	18.6	18.1	12–25.2	< .0001
Fever	4.8	5.2	0.4	5.1	3.9	1.6	1.2	-1.5–3.9	0.376
Prolonged labour	3.6	7.9	-4.3	7.4	7.6	-0.2	4.1	3.5–8.1	< .0001
Knowledge of at least 3 (Out of 10)	15.3	13.9	1.4	43.1	23.5	19.7	18.3	11.4–25.2	< .0001
During Postpartum									
Bleeding	15.1	19.0	-2.9	39.8	15.2	24.6	27.5	21.8–30.8	< .0001
Fever	2.2	5.3	-3.1	8.9	5.6	3.3	6.3	-1-6–5.2	0.763
Body weakness	7.8	4.2	3.6	4.2	3.5	0.7	-2.9	-1.1–6.8	0.145
Fits	8.9	6.6	2.3	16.5	7.2	9.3	7.0	2.3–15.1	< .0001
Knowledge of at least 3 (out of 8)	8.8	8.9	-0.1	19.8	9.2	9.3	9.4	6.4–15.7	< .0001

Table 4 shows the *knowledge* regarding the components of birth preparedness and complication readiness. The knowledge of three or more components of birth preparedness showed an improvement of 9.2 % (95 % CI: 2.8–13.2; *p* < .0001). Knowledge of identifying a blood donor improved with a net effect of 4.2 % (95 % CI: 2.0–6.5; *p* < .0001).

**Table 4:** Effect of the intervention on *knowledge* of birth preparedness and complication readiness among women in Rufiji and Mkuranga districts

	Pre intervention			Post intervention			Difference		
	*n* =798	*n* = 786		*n* = 744)	*n* = 742				
	Intervention (%)	Comparison (%)	Difference (%)	Intervention (%)	Comparison (%)	Difference (%)	NIE (%)	CI 95 %	*p*-value
Saving Money	36.5	39.6	-3.1	58.7	55.4	3.3	6.4	-3.5–16.4	0.195
Identify transport	6.3	4.2	2.1	22.0	6.0	16	13.9	3.8–27.4	< .0001
Identify Skilled attendant	0.3	0.5	-0.2	4.7	0.6	4.1	4.3	2.5–8.1	< .0001
Identify where to go	0.9	1.0	-0.1	10.5	0.9	9.6	9.7	3.3–17.9	0.035
Identify Blood donor	0.1	0.3	-0.2	4.5	0.5	4	4.2	2.0–6.5	< .0001
Identify Birth Kit	74.2	73.9	0.3	97.6	96.1	1.5	1.2	0.2–5.1	0.582
Knowledge of 3 BP/CR	2.3	2.8	-0.5	13.6	4.9	8.7	9.2	2.8–13.2	< .0001

Table 5 presents the *practice* of BP/CR among women during their last pregnancy before and after the intervention. Overall the intervention increased the proportion of women who took three or more steps in preparation for the birth with a two-fold increase, and net intervention effect of 13 % (95 % CI: 10.3–20.3; *p* < .0001). There was significant improvement on the proportion of women who identified a blood donor as part of their BP/CR: 3.7 % (95 % CI: 2.1–6.2; *p* < .0001).

**Table 5:** Effect of intervention of *practice* on birth preparedness and complication readiness among women in Rufiji and Mkuranga districts

	Pre intervention			Post intervention			Difference		
	*n* = 798	*n* = 786		*n* = 744	*n* = 742				
	Intervention (%)	Comparison (%)	Difference (%)	Intervention (%)	Comparison (%)	Difference (%)	NIE (%)	CI 95 %	*p-*value
Saved Money	66.5	67.4	-0.9	73.3	60.1	13.1	14	4.5–23.6	0.003
Identified Transport	21.9	31.8	-9.9	27.6	21.8	5.7	15.6	3.8–27.4	0.008
Identified Skilled attendant	1.3	1.1	-0.2	11.1	1.3	10.8	11	9.1–16.2	< .0001
Identified where to go	4.3	4.1	0.2	12.9	4.3	8.6	8.4	5.1–11.8	< .0001
Identified Blood donor	2.4	2.3	-0.1	3.9	0.3	3.6	3.7	2.1–6.2	< .0001
Identified Birth Kit	68.9	67.3	1.6	93.1	79.4	13.8	12.2	1.2–23.1	0.026
Practice of 3 BP/CR	20.8	23.5	-2.7	35.3	25.2	10.3	13	10.3–20.3	< .0001

## Discussion

This study shows that after training of the community on HBLSS more women delivered in health facilities, had more knowledge of danger signs and BP/CR and a larger proportion of women made at least three out of six birth preparations. There was also a significant improvement in the number of women who made four ANC visits as recommended in the Focused Antenatal Care (FANC) guidelines.

After the training, significantly more women could mention at least three dangers signs during pregnancy, delivery and the postpartum period respectively, similar to findings in other studies to determine improvement in knowledge of maternal danger signs in Eritrea, Kenya, Nepal and Bangladesh using CHWs as information guides [29–33]. Knowledge on bleeding during pregnancy and childbirth as well as prolonged labour showed significant improvement. It is encouraging that these signs are well known because they are the main causes of maternal mortality. Other studies have shown that providing counselling by CHWs on obstetric danger signs had an effect of increasing facility delivery [23, 34, 35]. Knowledge of danger signs is essential as evidence from other studies has shown that an increase in facility delivery can be explained by increased maternal knowledge of danger signs, as demonstrated in Zambia and Tanzania [36, 37].

We found that the number of four and more ANC visits increased significantly and adhered to the WHO-recommended number of visits. This improvement is promising as the national average for attending four ANC visits is 43 %. Community-based interventions from Eritrea and Bangladesh show a similar effect [32, 38]. ANC is important in that it provides an entry point between a pregnant woman and the health system. During the ANC visit, the woman is supposed to receive health education, immunization, be investigated for potential pre-existing problems, and counselled on danger signs and birth preparedness. All of this information is necessary in order to reduce delays in seeking care. It has also been shown that women initiate early booking at ANC care when they have been educated by safe motherhood promoters [10]. Furthermore, the number of ANC visits and knowledge of danger signs has been shown to influence the use of skilled care for delivery in Zambia and India [36, 39]. However, utilisation of skilled care for ANC can be challenging due to distance to the health facility, availability of services, cost related to accessing care, quality of care, and the woman’s socio-economic status [40–42]. A recent study performed in Tanzania to identify factors that hamper women making four visits argued that pregnant women should be encouraged to attend ANC early in their pregnancy in order to make the four recommended visits [40]. The use of CHWs to identify pregnant women early in their pregnancy, as done in this study, may have contributed to the increase in the number of ANC visits.

Birth preparedness messages are important in making a pregnant woman ready for normal or emergency delivery and help in reducing delays in seeking care. Both knowledge of birth preparedness as well as the actual preparation for childbirth, such as making transport arrangement and identifying a skilled birth attendant, increased in our study. Also the identification a blood donor improved significantly and this can be explained by the fact the community understands that bleeding is dangerous and can lead quickly to women’s deaths. This finding is similar to studies completed in rural Burkina Faso, Eritrea, Uganda and Kenya where the use of facility delivery and community-based interventions promoting BP/CR were associated with increased knowledge about BP/CR [29, 32, 43, 44]. This improvement can be attributed to the close interaction between CHWs and the community as compared to health workers who spend less time with women during ANC. In contrast, a cluster-randomised trial involving Argentina, Guatemala, Kenya, Zambia, India and Pakistan to improve pregnancy outcome using multiple interventions including HBLSS provided by health workers as one of the intervention component, did not show improvement in birth preparedness practices [45].

Facility-based delivery rates in the intervention area increased significantly from baseline to endline (75.6 vs. 90.2 %) while in the comparison area, they increased by 3 %. The increase in the intervention area was higher compared to the comparison area, but no net difference was observed. This could be explained by the fact that the facility delivery at this area was already at a higher level than expected from the Tanzania Demographic Health Survey that had a facility delivery rate of 47 % [25] and the sample size calculation was based on this information. The rate of 90.2 % is high compared to the national average of 50 % of facility delivery [12]. Other community intervention studies in the low-income countries that involved the use of CHWs in providing education and care in the community have also shown an increase in facility delivery [29, 38, 46–48]. Evidence shows that the use of maternal health services, including facility delivery, is associated with distance, living in a rural area, socio-economic status, maternal education and perception of quality of care at health facilities [49–53]. In this rural community it is encouraging that women now prefer facility delivery in spite of the barriers, as seen in most developing countries [54, 55]. Although our study did not assess the quality of care at facility level, the link between the health workers and CHWs during the training using culturally sensitive language may have contributed to the increase in facility delivery in the intervention area. Further research can illicit this linkage in improving the rate of facility delivery in a rural community. Our study signifies the role that CHWs can play in improving maternal health care utilization in rural areas. Supportive supervision by the health workers could have also contributed to the implementation of the work by CHWs. A recent study done in Morogoro to evaluate the supportive supervision of CHWs by health workers and village leaders in Integrated Maternal, Newborn and Child Health (MNCH) showed CHWs valued supervision and helped in improving their skills in providing education to community and problem solving [56]. The Tanzania government is planning to integrate the CHWs into the health system by paying their salaries; this will greatly contribute to the sustainability of the programme.

### Strengths and limitations

One of the strength of this study was the use of the same instrument to conduct the surveys pre/post and the use of a control district strengthens the interpretation of the results. CHWs were recruited from the study villages (insiders) and hence ownership and sustainability is feasible. The training of health workers could also have provided a sort of refresher course for them to improve their daily work. The large sample size and high response rate in the rural setting strengthen the study.

It is worth mentioning the limitations related to this study. The ability to fully interpret the results is limited to the nature of the pre/post design. A randomised controlled trial would have reduced the bias when interpreting the results. The data obtained also relied on the recall of the participants about events that occurred in the last two years. This would have introduced recall bias. Social desirability cannot be ruled out as the participants may have given responses that are thought to be the best desirable practices. Distance to health facility was not captured in the study and this is a limitation especially in utilization of skilled care.

## Conclusion

Our study has demonstrated that using an HBLSS training programme delivered by CHWs improved knowledge of danger signs, knowledge of birth preparedness and improved practice of BP/CR and facility delivery. ANC utilisation with four visits or more also improved. Although the net intervention effect did not show a statistically significant improvement in facility deliveries, there is some indication that this community-based intervention may have had an impact on this. CHWs could collaborate with health care workers in providing knowledge related to pregnancy and childbirth in order to improve ANC attendance and increase in rate of facility delivery. We suggest that a similar intervention using CHWs could be conducted in areas where facility delivery is low and hence improve skilled care utilisation and, ultimately, reduce maternal mortality.

## Abbreviations

ACNM, American College of Nurse Midwives; ANC, Antenatal Care; BP/CR, Birth Preparedness and Complication Readiness; CHWs, Community Health Workers; FANC, Focused Antenatal Care; HBLSS, Home Based Life Saving Skills; MDG, Millennium Development Goal; TAC, Take Action Card.

## Additional file

Additional file 1: *Home Based Life Saving Skills questionnaire, *Questionnaire used for the study. (PDF 160 kb)
