# Supplementary material for: Effectiveness of the Home Based Life Saving Skills training by community health workers on knowledge of danger signs, birth preparedness, complication readiness and facility delivery, among women in Rural Tanzania
Source: BMC Pregnancy Childbirth. 2016 Jun 2;16:129. doi: 10.1186/s12884-016-0916-x (PMC4890507; doi:10.1186/s12884-016-0916-x)
Supplement: Additional file 1: — *Home Based Life Saving Skills questionnaire, *Questionnaire used for the study. (PDF 160 kb) [file 12884_2016_916_MOESM1_ESM.pdf]

Questionnaire for Home Based Life Saving Skills

PART 1: SOCIO DEMOGRAPHIC CHARACTERISTICS.

Q #	Question	CODES
1.	How old are you now	**AGE IN COMPLETED YEARS…**
2.	Have you ever attended school?	**YES…………………………………………………...1**<br>**NO……………………………………………………2**
3.	What is the highest grade you have completed?	**NO SCHOOL……………………………………….1**<br>**NOT COMPLETED PRIMARY SCHOOL…………..2**<br>**COMPLETED PRIMARY SCHOOL………………….3**<br>**NOT COMPLETED SECONDARY SCHOOL……...4**<br>**COMPLETED SECONDARY SCHOOL……………5**<br>**UNIVERSITY……………………………………….6**<br>**MADRAS…………………………………………….7**<br>**OTHER…………………………………………….97**<br>**(SPECIFY)**
4.	What is your marital status now?	**SINGLE…………………………………………….....1**<br>**MARRIED/IN UNION………….……………………2**<br>**WIDOWED……………………………………………3**<br>**DIVORCED…………………………………………4**<br>**SEPARATED……………………………………….5**
5.	What is the main material of the floor<br><br>**Record observation**	**NATURAL FLOOR**<br>**EARTH/SAND ....11**<br>**DUNG...12**<br>**RUDIMENTARY FLOOR**<br>**WOOD PLANKS..21**<br>**PALM/BAMBOO ....22**<br>**FINISHED FLOOR**<br>**PARQUET OR**<br>**POLISHED WOOD.......................................31**<br>**VINYL OR**<br>**ASPHALT STRIPS..32**<br>**CERAMIC TILES..33**<br>**CEMENT...34**<br>**CARPET..35**<br>**OTHER...97**<br>**(SPECIFY)**
6.	What is the main source of drinking water for members of your household?	**PIPED WATER**<br>**PIPED INTO DWELLING ....11**<br>**PIPED INTO YARD/PLOT......................................12**<br>**PUBLIC TAP ....13**<br>**WATER FROM OPEN WELL**<br>**OPEN WELL IN YARD/PLOT ....21**<br>**OPEN PUBLIC WELL ……………………………....22**<br>**WATER FROM COVERED WELL PROTECTED WELL IN**<br>**YARD/PLOT ....31**<br>**PROTECTED PUBLIC WELL ....32**<br>**SURFACE WATER**<br>**SPRING...41**<br>**RIVER/STREAM..42**

		**POND/LAKE..43**<br>**DAM...44**<br>**RAINWATER ....51**<br>**TANKER TRUCK...61**<br>**BOTTLED WATER 71**
7.	What type of fuel do you mainly use for cooking in your household?	**ELECTRICITY...01**<br>**LPG/NATURAL GAS...02**<br>**BIOGAS..03**<br>**KEROSENE...04**<br>**CHARCOAL..05**<br>**FIREWOOD, STRAW ....06**<br>**DUNG...07**<br><br>**OTHER_______________________________ 97**<br>**(SPECIFY)**
8.	Does any member of your household own:	**YES NO**<br>**RADIO. ……………………………………1………2**<br>**CELL PHONE...1...........2**<br>**BICYCLE ....1...........2**<br>**MOTORCYCLE/SCOOTER.........................1...........2**<br>**CAR/TRUCK...1...........2**<br>**TRACTOR...1...........2**<br>**ANIMAL-DRAWN CART ....1...........2**
9.	How many children (both live and stillborn) did you give birth to<br>Alive now?	**No children yet_______________**<br><br>**Number of children__________**<br><br>**Number alive now?--------------**
10.	Have you ever given birth in the last 24 months, either to a baby that was born alive or a baby that was born dead?	**YES…………………………………………………………...1**<br>**NO……………………………………………………………2**
11.	Are you pregnant now?	**YES…………………..……………………………….…1**<br>**NO……………………………………………………………2**
12.	How many months pregnant are you?	**MONTHS…………………………………**
13.	Is this your first pregnancy	**YES…………………………………………………………...1**<br>**NO……………………………………………………………2**
14.	How many times did you attend ANC	**Number of ANC visits** ☐

PART 2: KNOWLEDGE

15.	Can unforeseen problems related to pregnancy occur that could endanger life or a woman?	**YES……01**<br>**NO……02**<br>**DON'T KNOW……98**
16.	What are some serious health problems that can occur during <u>pregnancy</u> , <u>childbirth</u> and <u>postpartum period</u> that could endanger the life of a pregnant woman<br><br>PROBE: Any others?	**BLEEDING……01**<br>**SEVERE HEADACHE……02**<br>**BLURRED ISION……03**<br>**CONVULSIONS……04**<br>**SWOLLEN HANDS/FACE……05**<br>**HIGH FEVER……06**<br>**LOSS OF CONSCIOUSNESS……07**<br>**DIFFICULTY BREATHING……08**<br>**SEVERE WEAKNESS……09**<br>**SEVERE ABDOMINAL PAIN……10**<br>**ACCELERATED/REDUCED FETAL MOVEMENT……11**<br>**WATER BREAKS WITHOUT LABOUR……12**<br><br>**OTHER________________97**<br>**(SPECIFY)**<br>**NONE……00**<br>**DON'T KNOW……98**<br><br>**<u>CHILDBIRTH</u>**<br>**SEVERE BLEEDING……01**<br>**SEVERE HEADACHE……02**<br>**CONVULSIONS……03**<br>**HIGH FEVER……04**<br>**LOSS OF CONSCIOUSNESS……05**<br>**LABOR LASTING>12HOURS……06**<br>**PLACENTA NOT DELIVERED 30 MINUTES AFTER BABY……07**<br><br>**OTHER________________97**<br>**(SPECIFY)**<br>**NONE……00**<br>**DON'T KNOW……98**<br><br>**<u>POSTPARTUM</u>**<br>**SEVERE BLEEDING……01**<br>**SEVERE HEADACHE……02**<br>**CONVULSIONS……03**<br>**HIGH FEVER……04**<br>**LOSS OF CONSCIOUSNESS……05**<br>**LABOR LASTING>12HOURS……06**<br>**MALODOROUS VAGINAL DISCHARGE……07**<br>**DIFFICULTY BREATHING……08**<br>**SEVERE WEAKNESS……09**<br>**OTHER________________97**<br>**(SPECIFY)**<br>**NONE……00**<br>**DON'T KNOW……98**

17.	In your opinion, what are some serious problems that can occur during the first 7 days after birth that could endanger the life of the new born baby?	**DIFFICULT OR FAST BREATHING……………..……..01**<br>**YELLOW SKIN/EYE COLOR (JAUNDICE)……...…02**<br>**POOR SUCKING OR FEEDING……………………..…..03**<br>**PUS, BLEEDING OR DISCHARGE FROM THE UMBILICAL CORD…………………………...…..04**<br>**BABY VERY SMALL……………………………..…..05**<br>**SKIN LESIONS OR BLISTERS…………………..……06**<br>**CONVULSIONS/SPASMS/RIGIDITY……………..….07**<br>**ABDOMINAL PAIN/FULLNESS…………………..…..08**<br>**FAILURE TO PASS STOOL……………………..……09**<br>**FEVER…………………………………………………10**<br>**OTHER…………………………………………..…..97**<br>**(SPECIFY)**<br>**DON'T KNOW………………………………………...00**<br>**NONE…………………………………………………..98**
18.	Have you ever heard the term 'birth preparedness'?	**YES……………………….…………………………..1**<br>**NO…………………………………………………….2**
19.	In your opinion, what are some things a woman/family can do to prepare for birth?	**IDENTIFY MODE OF TRANSPORT...........................01**<br>**SAVE MONEY (for emergencies)...................................02**<br>**IDENTIFY BLOOD DONOR ....03**<br>**IDENTIFY SKILLED PROVIDER...............................04**<br>**IDENTIFY WHERE TO GO…………………………....05**<br>**BIRTH KIT……………………………………………...06**<br>**OTHER ______________________ 97**<br>**(SPECIFY)**
20.	In your community, which is the commonest mean of transportation to use to go to your nearest health facility<br><br>PROBE:	**AMBULANCE…………………………………………....01**<br>**PRIVATE CAR………...………………………..…..02**<br>**TAX/BUS…………………………………………..…..03**<br>**CART……………………………………………..……04**<br>**MOTORBIKE……………………………………………05**<br>**ON FOOT…………………………………………..……06**<br>**BICYCLE………………………………………………...07**<br><br>**OTHER______________________97**<br>**(SPECIFY)**<br>**DON'T KNOW…………………………………………98**
21.	In general, how long would it take to reach this health facility?	**HOURS…………………………** ☐<br><br>**MINUTES………………………………** ☐<br><br>**DON'T KNOW…………………………………………..98**

22.	**ATTITUDES AND PERCEPTION**<br>A woman should plan ahead of time where she will give birth to hear baby	**SA**<br>**01**	**A**<br>**02**	**D**<br>**03**	**SD**<br>**04**	**DK**<br>**05**

23.	A woman should plan ahead of time how she will get to the place where she will give birth	**SA 01**	**A 02**	**D 03**	**SD 04**	**DK 05**
24.	It is not necessary for a husband /partner to accompany his wife to antenatal care visits	**SA 01**	**A 02**	**D 03**	**SD 04**	**DK 05**
25.	It is not necessary for a husband to accompany his wife when she is giving birth	**SA 01**	**A 02**	**D 03**	**SD 04**	**DK 05**
26.	Giving birth is mostly woman's matter. Husbands have little to contribute	**SA 01**	**A 02**	**D 03**	**SD 04**	**DK 05**

PART 3: PERSONAL EXPERIENCE RELATED TO THIS OR THE LAST PREGNANCY.

Q. #	QUESTION	CODES
27.	During this or the last pregnancy, did a health worker advise you about any of the following at least once?	**YES NO DK**<br>**DANGER SIGNS……………......01….......02………....98**<br>**WHERE TO GO ……………......01…........02……......98**<br>**WHERE TO GIVE BIRTH…………………......01…......02……......98**<br>**TRANSPORT ...……………....01…......02……......98**<br>**MONEY ...……………………......01…......02……......98**<br>**BLOOD DONOR………………..01…......02……......98**<br>**BIRTH KIT…………………...…01…...…02……......98**
28.	Did you speak with anyone, outside of a health facility about danger signs of serious health problems during pregnancy, childbirth, or soon after?<br><br>IF YES: Who did you speak with?<br><br>RECORD ALL RESPONSES.	**YES NO DK**<br>**DANGER SIGNS……………...01…......02……......98**<br>**HUSBAND ……………………………………..…01**<br>**MOTHER-IN-LAW……………..…………………02**<br>**OTHER FAMILY MEMBER……….……………03**<br>**FRIEND/NEIGHBOR……………………..…………04**<br>**COMMUNITY HEALTH WORKER………….…05**
29.	Did you speak with anyone outside of a health facility about danger signs of serious health problems for your baby<br>IF YES: Who did you speak with?<br><br>RECORD ALL RESPONSES.	**YES NO DK**<br>**DANGER SIGNS…..................01…......02……......98**<br>**HUSBAND ………………………..…..………01**<br>**MOTHER-IN-LAW……………………….…………02**<br>**OTHER FAMILY MEMBER…………………03**<br>**FRIEND/NEIGHBOR……………………….…04**<br>**COMMUNITY HEALTH WORKER…………05**

30.	Did you speak with anyone outside of a health facility about <u>Where to go if you had danger signs of serious health problems</u>?<br><br>IF YES: Who did you speak with?<br><br>RECORD ALL RESPONSES.	**YES NO DK**<br>**WHERE TO GO……………....01……..02……......98**<br>**HUSBAND ……………………………………..01**<br>**MOTHER-IN-LAW…………………….……..02**<br>**OTHER FAMILY MEMBER……………………03**<br>**FRIEND/NEIGHBOR……………………….…04**<br>**COMMUNITY HEALTH WORKER……………05**
	<u>Where should give birth</u>	**YES NO DK**<br>**WHERE TO GIVE BIRTH ………………...01……..02…….....98**<br>**HUSBAND……………………………………...01**<br>**MONTHER-IN-LAW…………………….……..02**<br>**OTHER FAMILY MEMBER………..…..……...03**<br>**FRIEND/NEIGHBOR…………………......…04**<br>**COMMUNITY HEALTH WORKER……………05**
	<u>Arrangement of transport</u>	**YES NO DK**<br>**TRANSPORT ………………...01……..02…….....98**<br>**HUSBAND……………………………………..01**<br>**MONTHER-IN-LAW…………………...……02**<br>**OTHER FAMILY MEMBER……………….……03**<br>**FRIEND/NEIGHBOR…………….…….….…04**<br>**COMMUNITY HEALTH WORKER..………...05**
	<u>Arrangement for funds/finances?</u>	**YES NO DK**<br>**MONEY …………………….....01……..02…….....98**<br>**HUSBAND……………………………………..01**<br>**MONTHER-IN-LAW……………………………02**<br>**OTHER FAMILY MEMBER…………..……03**<br>**FRIEND/NEIGHBOR……………………..…..…04**<br>**COMMUNITY HEALTH WORKER………..…05**
	<u>Arrangement for blood donor?</u>	**YES NO DK**<br>**BLOOD DONOR …………….01……..02…….....98**<br>**HUSBAND…………………….……………..….01**<br>**MONTHER-IN-LAW…………………………..…02**<br>**OTHER FAMILY MEMBER…………..….…...03**<br>**FRIEND/NEIGHBOR…………………………..…04**<br>**COMMUNITY HEALTH WORKER……………05**

31.	Did you speak with anyone outside of a health facility about arrangement for a healthcare professional to deliver your child?<br><br>IF YES: Who did you speak with?<br><br>RECORD ALL RESPONSES.	**YES NO DK**<br>**SKILLED PROVIDER ………..01…….02…......98**<br>**HUSBAND…………………………..………..01**<br>**MONTHER-IN-LAW……………….……….02**<br>**OTHER FAMILY MEMBER……………..…..03**<br>**FRIEND/NEIGHBOR……………………..….04**<br>**COMMUNITY HEALTH WORKER……….05**
32.	Have you or your family made any arrangements for the birth of this child?	**YES…..……………………………….….….……….01**<br>**NO………………………………….……………..……..02**<br>**DON'T KNOW…………………………….………….98**
33.	Which arrangements have you or your family made for the birth of this child?	**UNPROMPTED PROMPTED**<br>**IDENTIFY**<br>**TRANSPORT………………..01…………………01**<br>**SAVE MONEY………………...02……………..…02**<br>**IDENTIFY BLOOD**<br>**DONOR…………………03……………..….03**<br>**IDENTIFY SKILLED**<br>**PROVIDER…………………04…………………04**<br>**IDENTIFY WHERE TO GO…05…………………05**<br>**BIRTH KIT……………………06…………………06**<br><br>**OTHER________________________________97**<br>**(SPECIFY)**
34.	Where do you plan to give birth to this baby?	**HOME**<br>**RESPONDENT'S HOME……….……….……....11**<br>**TBA'S HOME……………………………………..12**<br>**OTHER HOME……..…………….…………..…..13**<br><br>**PUBLIC SECTOR**<br>**GVT. HOSPITAL……………………......…….21**<br>**GVT. HEALTH CENTER……………..………..…22**<br>**GVT. DISPENSARY………………...…………23**<br>**OTHERPUBLIC__________________________26**<br>**(SPECIFY)**<br><br>**PRIVATE SECTOR**<br>**PVT. HOSPITAL…………………………………31**<br>**MATERNITY/NURSING HOME……..……...32**<br>**OTHER PRIVATE _______________________36**<br>**(SPECIFY)**<br><br>**OTHER ______________________________97**<br>**(SPECIFY)**

PART 4: EXPERIENCE RELATED TO THIS OR THE LAST PREGNANCY

Q. #	QUESTION	CODES
35.	During this or the last pregnancy, did you experience any serious health problems related to the pregnancy?	**YES …………01**<br>**NO…………02**<br>**DON'T KNOW…………98**
36.	What problems did you experience? (CIRCLE ALL RESPONSES GIVEN).<br><br>THEN PROBE: Did you experience (ANY REMAINING COMPLICATIONS)?	**UNPROMPTED PROMPTED**<br>**BLEEDING…………01…………01**<br>**SEVERE HEADACHE. …………02…………02**<br>**BLURRED VISION …………03…………03**<br>**CONVULSIONS …………04…………04**<br>**SWOLLEN HANDS/FACE…………05…………05**<br>**HIGH FEVER…………06…………06**<br>**LOSS OF CONSCIOUSNESS… 07…………07**<br>**DIFFICULTY BREATHING…...08…………08**<br>**SEVERE WEAKNESS…………09…………09**<br>**SEVERE ABDOMINAL PAIN…………10…………10**<br>**ACCELERATED/REDUCED FETAL MOVEMENT…………11…………11**<br>**WATER BREAKS WITHOUT LABOR…………12…………12**<br><br>**OTHER________________97**<br>**(SPECIFY)**<br><br>**DON'T KNOW…………98**
37.	Did you seek assistance for this problem?	**YES …………01**<br>**NO…………02**<br>**DON'T KNOW…………98**
38.	IF YES: Where did you got?<br><br>________________<br>(NAME OF PLACE)	**PUBLIC SECTOR**<br>**GVT. HOSPITAL…………21**<br>**GVT. HEALTH CENTER…………22**<br>**GVT. DISPENSARY…………23**<br><br>**OTHER PUBLIC ____________26**<br>**(SPECIFY)**<br>**PRIVATE SECTOR**<br>**PVT. HOSPITAL…………31**<br>**MATERNITY/NURSING HOME…………32**<br>**OTHER PRIVATE ____________36**<br>**(SPECIFY)**<br><br>**OTHER ________________97**<br>**(SPECIFY)**

Q. #	QUESTION	CODES
39.	Why did you not seek assistance for this problem?<br><br>Anything else?<br><br>PROBE FOR OTHER REASONS AND RECORD ALL REASONS MENTIONED.	**RESP. DIDN'T THINK NECESSARY……………..01**<br>**HUSBAND /FAMILY DIDN'T**<br>**THINK NECESSARY……………..…………..02**<br>**FACILITY TOO FAR………………………………..03**<br>**NOT TRANSPORT…………………………………..04**<br>**NO CHILDCARE……………………………………05**<br>**TOO EXPENSIVE…………………………………..06**<br>**SERVICES ARE POOR………………...…………07**<br>**USED HOME REMEDY……………………………..08**<br>**DID NOT KNOW WHERE TO GO…………….….09**<br>**NO TIME TO GO…………………………….…..10**<br><br>**OTHER____________________________97**<br>**(SPECIFY)**<br>**DON'T KNOW………………………………………98**
40.	Who made the final decision about whether or not to seek assistance for this problem?	**NO ONE…………………………………………….01**<br>**RESPONDENT……………………………………….02**<br>**RESPONDENT & HUSBAND……………………….03**<br>**HUSBAND…………………………………………….04**<br>**RESP'S MOTHER…………………………………….05**<br>**RESP'S FATHER…………………………………….06**<br>**MOTHER IN LAW…………………………………….07**<br>**FATHER IN LAW……………………………………08**<br>**FRIEND/NEIGHBOUR……………………………...09**<br>**HEALTH PROFESSIONAL………………………...10**<br>**TBA…………………………………………………11**<br><br>**OTHER____________________________97**<br>**(SPECIFY)**<br>**DON'T KNOW……………………………………..98**

PART 5: PERSONAL EXPERIENCE RELATED TO LAST BIRTH.

Q. #	QUESTION	CODES
41.	Where did you give birth to your last child?<br><br>____________________<br>(NAME OF PLACE)	**HOME**<br>**RESPONDENT'S HOME……….………....11**<br>**TBA'S HOME……………………………..12**<br>**OTHER HOME…….……………………..13**<br><br>**PUBLIC SECTOR**<br>**GVT. HOSPITAL……………………..……21**<br>**GVT. HEALTH CENTER…………….……22**<br>**GVT. DISPENSARY…………………..……23**<br>**OTHERPUBLIC__________________26**<br>**(SPECIFY)**<br><br>**PRIVATE SECTOR**<br>**PVT. HOSPITAL……………………………31**<br>**MATERNITY/NURSING HOME…..……...32**<br>**OTHER PRIVATE _______________36**<br>**(SPECIFY)**<br><br>**OTHER ________________________97**<br>**(SPECIFY)**
42.	Did you plan to give birth at this place?	**YES……………………………………………..01**<br>**NO………………………………………………02**<br>**DON'T KNOW……………..…………………98**
43.	Prior to this birth, did you or your family make any arrangements for the birth of this child?	**YES……………………………………………..01**<br>**NO………………………………………………02**<br>**DON'T KNOW……………..…………………98**
44.	Which arrangements did you or your family make for the birth of this child?<br>(CIRCLE ALL RESPONSES GIVEN.)<br>THEN PROBE: Did you [ANY REMAINING ARRANGEMENTS]?	**UNPROMPTED PROMPTED**<br>**IDENTIFY**<br>**TRANSPORT................01...........................01**<br>**SAVE MONEY**<br>**(for emergencies).......... 01............................02**<br>**IDENTIFY BLOOD**<br>**DONOR.......................03..........................03**<br>**IDENTIFY SKILLED**<br>**PROVIDER………04………………04**<br>**IDENTIFY**<br>**WHERE TO GO ………05……………..05**<br>**BIRTH KIT………………06………………..06**<br>**OTHER ______________________ 97**<br>**(SPECIFY)**
45.	Did you use the mode of transport that you identified?	**YES..01**<br>**NO..02**<br>**DON'T KNOW..98**
46.	Did you use the money saved for pregnancies emergencies or child emergencies?	**YES..01**<br>**NO..02**<br>**DON'T KNOW..98**
47.	Did you use the blood donor you identified	**YES..01**<br>**NO..02**<br>**DON'T KNOW..98**

48.	How did you go to the health facility?<br>PROBE: What type of transportation did you mainly use to get to the health facility?	**AMBULANCE...01**<br>**PRIVATE CAR..02**<br>**TAXI/BUS..03**<br>**CART...04**<br>**MOTORBIKE..05**<br>**ON FOOT ....06**<br>**BICYCLE...07**<br>**OTHER _______________________________ 97**<br>**(SPECIFY)**<br>**DON'T KNOW...98**
49.	Who accompanied you to the place where you gave birth?<br>PROBE FOR THE PERSON(S) ACCOMPANYING AND RECORD ALL PERSONS.	**NO ONE..01**<br>**HUSBAND...02**<br>**RESP.'S MOTHER..03**<br>**RESP.'S FATHER...04**<br>**MOTHER-IN-LAW...05**<br>**FATHER-IN-LAW ....06**<br>**SISTER/SISTER-IN-LAW.................................07**<br>**OTHER MEMBER OF RESP.'S FAM.............08**<br>**OTHER MEMBER OF HUSB.'S FAM ....09**<br>**FRIEND/NEIGHBOR..10**<br>**COMMUNITY HEALTH WORKER................11**<br>**TBA...12**<br>**OTHER _______________________________ 97**<br>**(SPECIFY)**<br>**DON'T KNOW 98**
50.	Can you tell me why you did not give birth in a health facility	**RESP. DIDN'T THINK NECESSARY..............01**<br>**HUSBAND/FAMILY DIDN'T**<br>**THINK NECESSARY...02**<br>**FACILITY TOO FAR..03**<br>**NO TRANSPORT..04**<br>**NO CHILDCARE..05**<br>**TOO EXPENSIVE...06**<br>**SERVICES ARE POOR.....................................07**<br>**DID NOT KNOW WHERE TO GO...................08**<br>**NO TIME TO GO..09**<br>**OTHER _______________________________ 97**<br>**(SPECIFY)**<br>**DON'T KNOW 98**
51.	Who assisted with the birth?	**HEALTH PROFESSIONAL……………..……….01**<br>**TBA...02**<br>**COMMUNITY HEALTH WORKER................03**<br>**RELATIVE/FRIEND ....04**<br>**OTHER ________________________________97**<br>**(SPECIFY)**
52.	Have you seen, heard or read any information regarding birth preparedness in the past sex months	**YES ………………………………...………...….01**<br>**NO……………………………………………..……..02**<br>**DON'T REMEMBER…………………………….98**
53.	From which source did you see hear or read about birth preparedness?<br>(CIRCLE ALL RESPONSES GIVEN)	**RADIO……………………………………………………………01**<br>**TV……………………………………………………..……………02**<br>**WRITTEN SOURCES……………………………...…………03**<br>**INTERPERSONAL SOURCES…………………...…………04**<br>**OTHER____97**<br>**(SPECIFY)**<br>**DON'T REMEMBER……………………………………………98**

54.	Have you participated in any community activities related to birth preparedness in the past six months	**YES ……………………………...………...…01**<br>**NO…………………………………………..……..02**<br>**DON'T REMEMBER.……………………….…..98**
55.	Through which activities did you hear about birth preparedness(CIRCLE ALL RESPONSES GIVEN)	**STREET DRAMA…………………………………...01**<br>**COMMUNITY MEETINGS………………………..02**<br>**MOTHER'S GROUPS………………………………03**<br>**OTHER________________________________97**<br>**(SPECIFY)**
